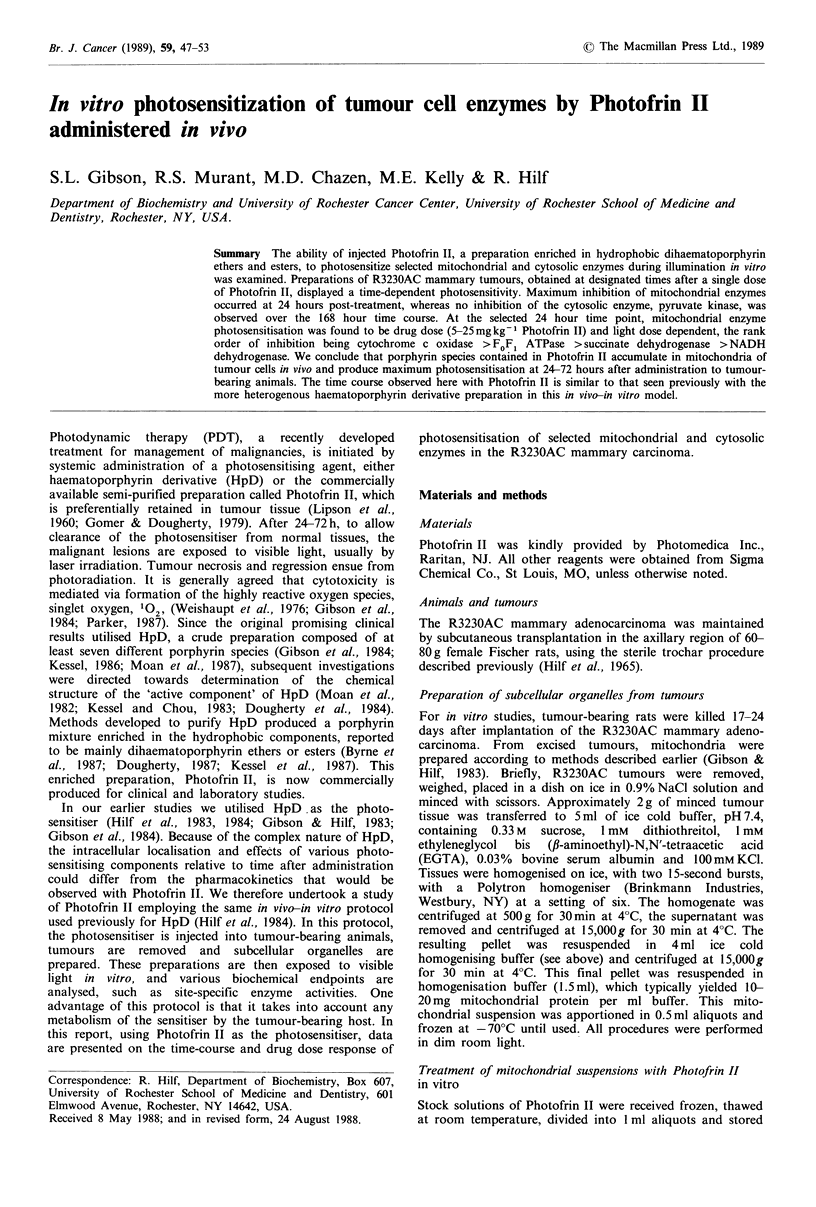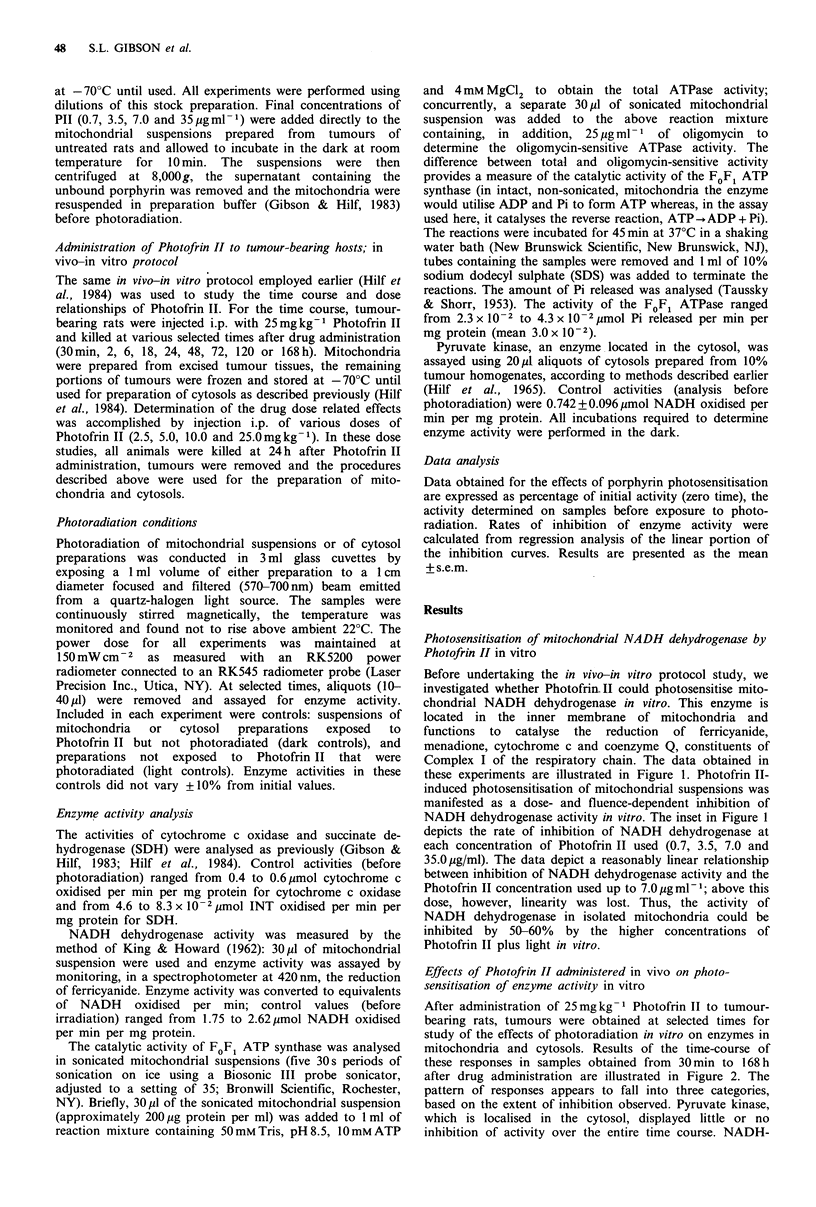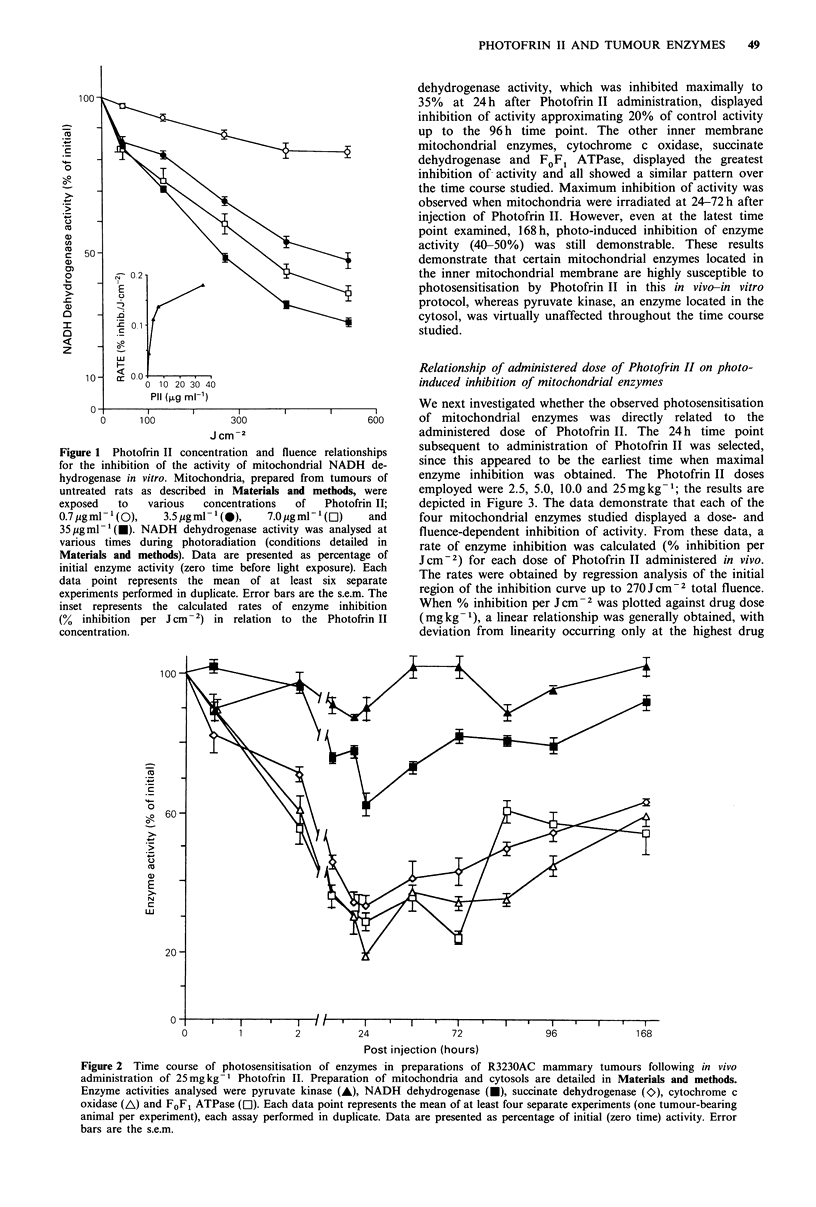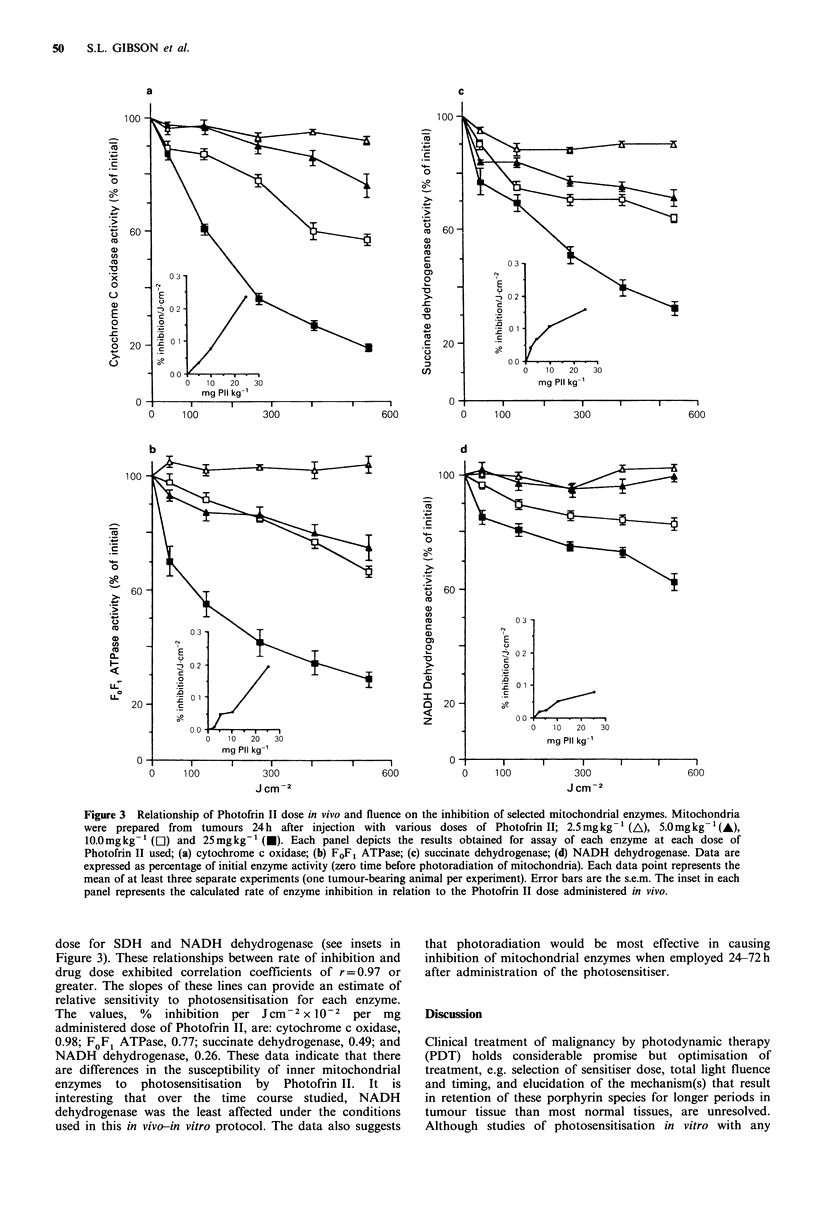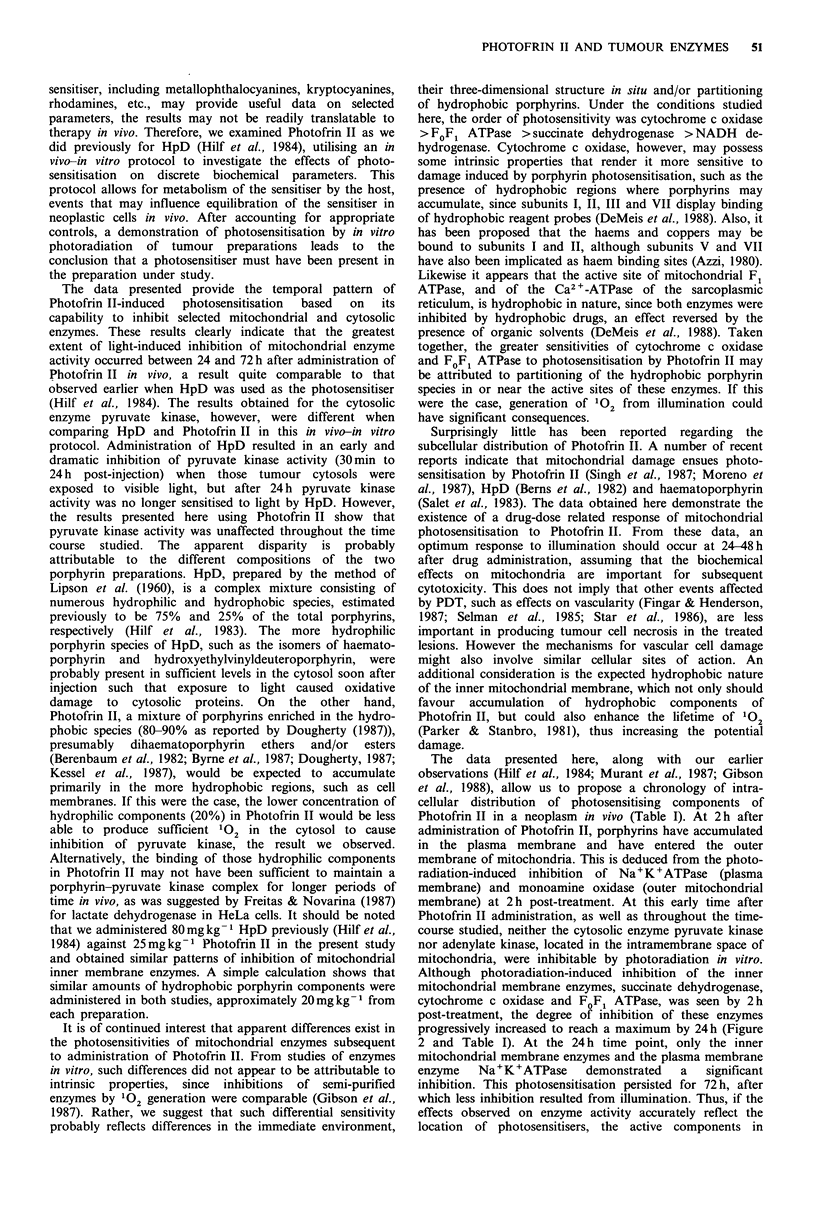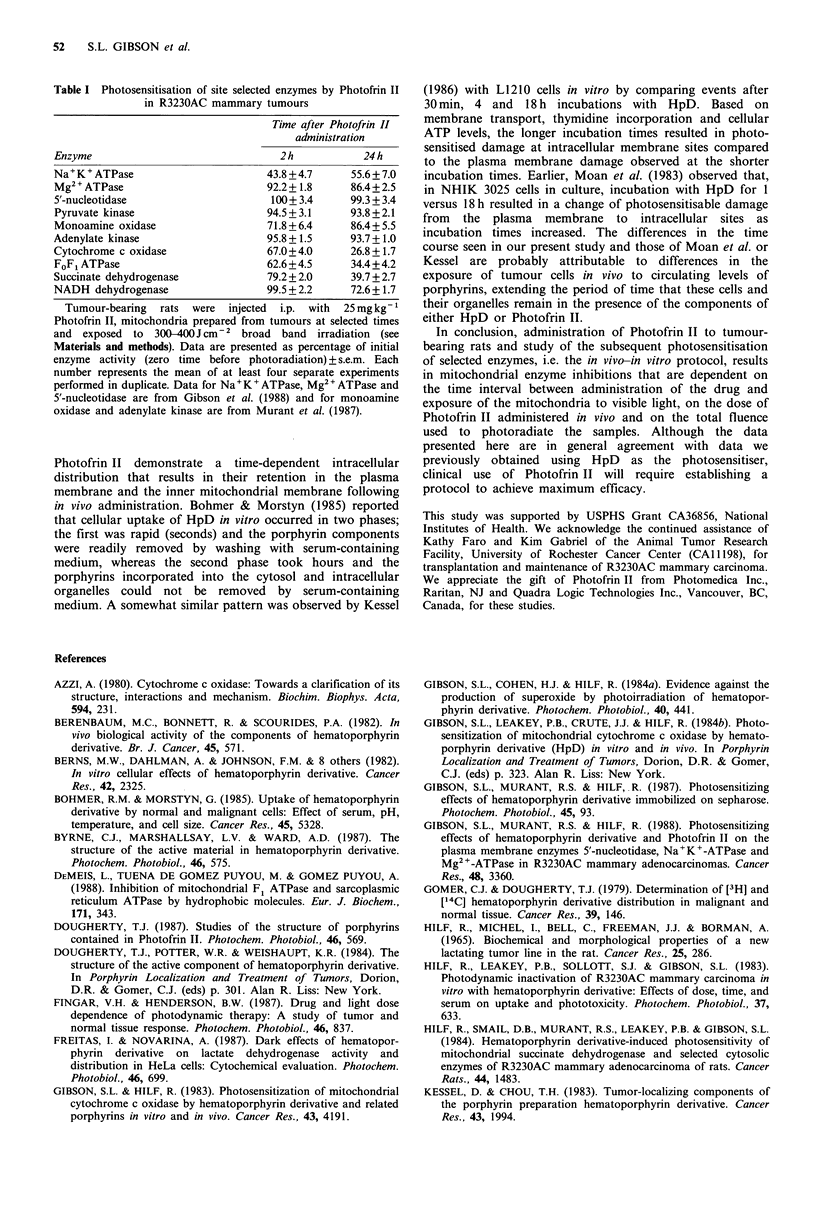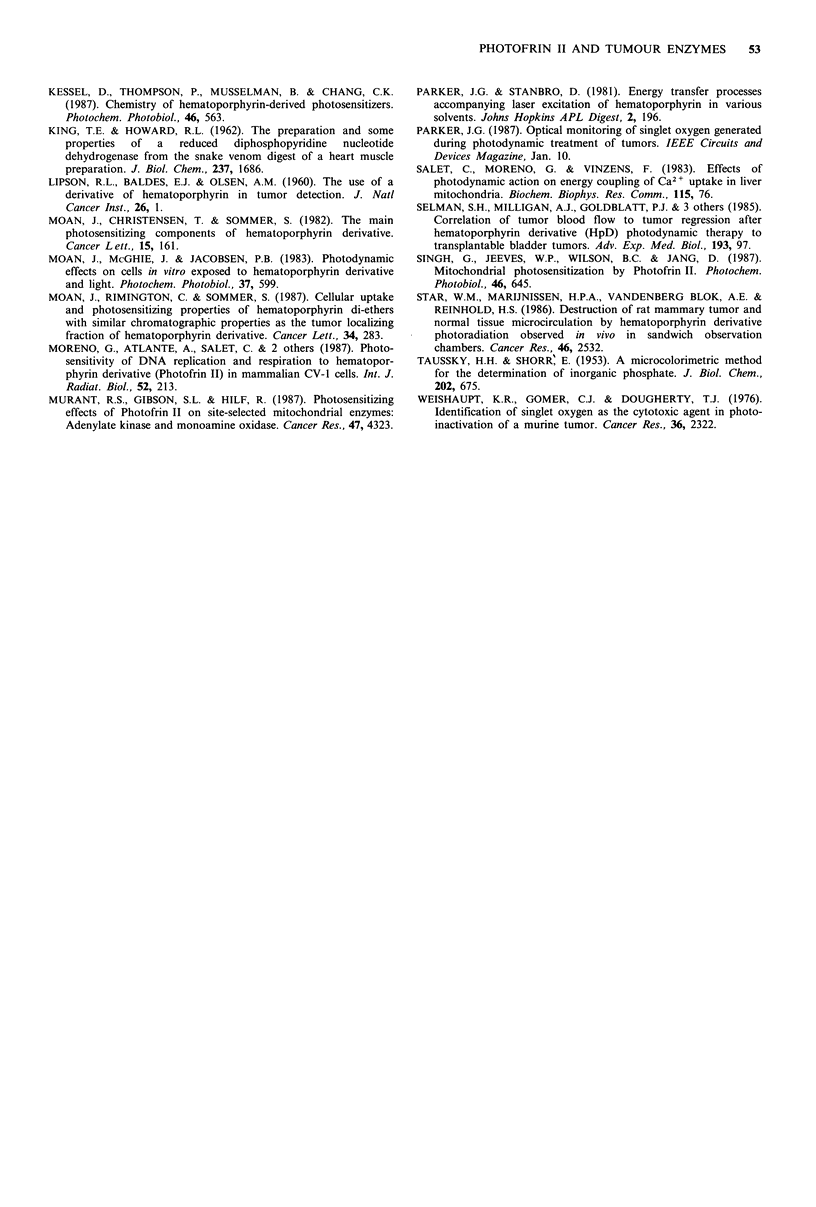# In vitro photosensitization of tumour cell enzymes by photofrin II administered in vivo.

**DOI:** 10.1038/bjc.1989.10

**Published:** 1989-01

**Authors:** S. L. Gibson, R. S. Murant, M. D. Chazen, M. E. Kelly, R. Hilf

**Affiliations:** Department of Biochemistry, University of Rochester School of Medicine and Dentistry, NY 14642.

## Abstract

The ability of injected Photofrin II, a preparation enriched in hydrophobic dihaematoporphyrin ethers and esters, to photosensitize selected mitochondrial and cytosolic enzymes during illumination in vitro was examined. Preparations of R3230AC mammary tumours, obtained at designated times after a single dose of Photofrin II, displayed a time-dependent photosensitivity. Maximum inhibition of mitochondrial enzymes occurred at 24 hours post-treatment, whereas no inhibition of the cytosolic enzyme, pyruvate kinase, was observed over the 168 hour time course. At the selected 24 hour time point, mitochondrial enzyme photosensitisation was found to be drug dose (5.25 mg kg-1 Photofrin II) and light dose dependent, the rank order of inhibition being cytochrome c oxidase greater than F0F1 ATPase greater than succinate dehydrogenase greater than NADH dehydrogenase. We conclude that porphyrin species contained in Photofrin II accumulate in mitochondria of tumour cells in vivo and produce maximum photosensitisation at 24-72 hours after administration to tumour-bearing animals. The time course observed here with Photofrin II is similar to that seen previously with the more heterogenous haematoporphyrin derivative preparation in this in vivo-in vitro model.


*Br. J. Cancer* (1989), **59**, 47–53 © The Macmillan Press Ltd., 1989

### *In vitro* photosensitization of tumour cell enzymes by Photofrin II administered *in vivo*

S.L. Gibson, R.S. Murant, M.D. Chazen, M.E. Kelly & R. Hilf

*Department of Biochemistry and University of Rochester Cancer Center, University of Rochester School of Medicine and Dentistry, Rochester, NY, USA.*

**Summary** The ability of injected Photofrin II, a preparation enriched in hydrophobic dihaematoporphyrin ethers and esters, to photosensitize selected mitochondrial and cytosolic enzymes during illumination *in vitro* was examined. Preparations of R3230AC mammary tumours, obtained at designated times after a single dose of Photofrin II, displayed a time-dependent photosensitivity. Maximum inhibition of mitochondrial enzymes occurred at 24 hours post-treatment, whereas no inhibition of the cytosolic enzyme, pyruvate kinase, was observed over the 168 hour time course. At the selected 24 hour time point, mitochondrial enzyme photosensitisation was found to be drug dose (5–25 mg kg$^{-1}$ Photofrin II) and light dose dependent, the rank order of inhibition being cytochrome c oxidase >$F_0F_1$ ATPase >succinate dehydrogenase >NADH dehydrogenase. We conclude that porphyrin species contained in Photofrin II accumulate in mitochondria of tumour cells *in vivo* and produce maximum photosensitisation at 24–72 hours after administration to tumour-bearing animals. The time course observed here with Photofrin II is similar to that seen previously with the more heterogenous haematoporphyrin derivative preparation in this *in vivo–in vitro* model.

Photodynamic therapy (PDT), a recently developed treatment for management of malignancies, is initiated by systemic administration of a photosensitising agent, either haematoporphyrin derivative (HpD) or the commercially available semi-purified preparation called Photofrin II, which is preferentially retained in tumour tissue (Lipson *et al.*, 1960; Gomer & Dougherty, 1979). After 24–72 h, to allow clearance of the photosensitiser from normal tissues, the malignant lesions are exposed to visible light, usually by laser irradiation. Tumour necrosis and regression ensue from photoradiation. It is generally agreed that cytotoxicity is mediated via formation of the highly reactive oxygen species, singlet oxygen, $^1O_2$, (Weishaupt *et al.*, 1976; Gibson *et al.*, 1984; Parker, 1987). Since the original promising clinical results utilised HpD, a crude preparation composed of at least seven different porphyrin species (Gibson *et al.*, 1984; Kessel, 1986; Moan *et al.*, 1987), subsequent investigations were directed towards determination of the chemical structure of the 'active component' of HpD (Moan *et al.*, 1982; Kessel and Chou, 1983; Dougherty *et al.*, 1984). Methods developed to purify HpD produced a porphyrin mixture enriched in the hydrophobic components, reported to be mainly dihaematoporphyrin ethers or esters (Byrne *et al.*, 1987; Dougherty, 1987; Kessel *et al.*, 1987). This enriched preparation, Photofrin II, is now commercially produced for clinical and laboratory studies.

In our earlier studies we utilised HpD as the photosensitiser (Hilf *et al.*, 1983, 1984; Gibson & Hilf, 1983; Gibson *et al.*, 1984). Because of the complex nature of HpD, the intracellular localisation and effects of various photosensitising components relative to time after administration could differ from the pharmacokinetics that would be observed with Photofrin II. We therefore undertook a study of Photofrin II employing the same *in vivo–in vitro* protocol used previously for HpD (Hilf *et al.*, 1984). In this protocol, the photosensitiser is injected into tumour-bearing animals, tumours are removed and subcellular organelles are prepared. These preparations are then exposed to visible light *in vitro*, and various biochemical endpoints are analysed, such as site-specific enzyme activities. One advantage of this protocol is that it takes into account any metabolism of the sensitiser by the tumour-bearing host. In this report, using Photofrin II as the photosensitiser, data are presented on the time-course and drug dose response of photosensitisation of selected mitochondrial and cytosolic enzymes in the R3230AC mammary carcinoma.

Correspondence: R. Hilf, Department of Biochemistry, Box 607, University of Rochester School of Medicine and Dentistry, 601 Elmwood Avenue, Rochester, NY 14642, USA.
Received 8 May 1988; and in revised form, 24 August 1988.

### Materials and methods

### *Materials*

Photofrin II was kindly provided by Photomedica Inc., Raritan, NJ. All other reagents were obtained from Sigma Chemical Co., St Louis, MO, unless otherwise noted.

### *Animals and tumours*

The R3230AC mammary adenocarcinoma was maintained by subcutaneous transplantation in the axillary region of 60–80 g female Fischer rats, using the sterile trochar procedure described previously (Hilf *et al.*, 1965).

### *Preparation of subcellular organelles from tumours*

For *in vitro* studies, tumour-bearing rats were killed 17–24 days after implantation of the R3230AC mammary adenocarcinoma. From excised tumours, mitochondria were prepared according to methods described earlier (Gibson & Hilf, 1983). Briefly, R3230AC tumours were removed, weighed, placed in a dish on ice in 0.9% NaCl solution and minced with scissors. Approximately 2 g of minced tumour tissue was transferred to 5 ml of ice cold buffer, pH 7.4, containing 0.33 M sucrose, 1 mM dithiothreitol, 1 mM ethyleneglycol bis (β-aminoethyl)-N,N′-tetraacetic acid (EGTA), 0.03% bovine serum albumin and 100 mM KCl. Tissues were homogenised on ice, with two 15-second bursts, with a Polytron homogeniser (Brinkmann Industries, Westbury, NY) at a setting of six. The homogenate was centrifuged at 500 g for 30 min at 4°C, the supernatant was removed and centrifuged at 15,000 *g* for 30 min at 4°C. The resulting pellet was resuspended in 4 ml ice cold homogenising buffer (see above) and centrifuged at 15,000 *g* for 30 min at 4°C. This final pellet was resuspended in homogenisation buffer (1.5 ml), which typically yielded 10–20 mg mitochondrial protein per ml buffer. This mitochondrial suspension was apportioned in 0.5 ml aliquots and frozen at −70°C until used. All procedures were performed in dim room light.

### *Treatment of mitochondrial suspensions with Photofrin II* in vitro

Stock solutions of Photofrin II were received frozen, thawed at room temperature, divided into 1 ml aliquots and stored at $-70°C$ until used. All experiments were performed using dilutions of this stock preparation. Final concentrations of PII (0.7, 3.5, 7.0 and $35\,\mu g\,ml^{-1}$) were added directly to the mitochondrial suspensions prepared from tumours of untreated rats and allowed to incubate in the dark at room temperature for 10 min. The suspensions were then centrifuged at 8,000 **g**, the supernatant containing the unbound porphyrin was removed and the mitochondria were resuspended in preparation buffer (Gibson & Hilf, 1983) before photoradiation.

*Administration of Photofrin II to tumour-bearing hosts;* in vivo–in vitro *protocol*

The same *in vivo–in vitro* protocol employed earlier (Hilf *et al.,* 1984) was used to study the time course and dose relationships of Photofrin II. For the time course, tumour-bearing rats were injected i.p. with $25\,mg\,kg^{-1}$ Photofrin II and killed at various selected times after drug administration (30 min, 2, 6, 18, 24, 48, 72, 120 or 168 h). Mitochondria were prepared from excised tumour tissues, the remaining portions of tumours were frozen and stored at $-70°C$ until used for preparation of cytosols as described previously (Hilf *et al.,* 1984). Determination of the drug dose related effects was accomplished by injection i.p. of various doses of Photofrin II (2.5, 5.0, 10.0 and $25.0\,mg\,kg^{-1}$). In these dose studies, all animals were killed at 24 h after Photofrin II administration, tumours were removed and the procedures described above were used for the preparation of mitochondria and cytosols.

*Photoradiation conditions*

Photoradiation of mitochondrial suspensions or of cytosol preparations was conducted in 3 ml glass cuvettes by exposing a 1 ml volume of either preparation to a 1 cm diameter focused and filtered (570–700 nm) beam emitted from a quartz-halogen light source. The samples were continuously stirred magnetically, the temperature was monitored and found not to rise above ambient 22°C. The power dose for all experiments was maintained at $150\,mW\,cm^{-2}$ as measured with an RK5200 power radiometer connected to an RK545 radiometer probe (Laser Precision Inc., Utica, NY). At selected times, aliquots (10–40 $\mu l$) were removed and assayed for enzyme activity. Included in each experiment were controls: suspensions of mitochondria or cytosol preparations exposed to Photofrin II but not photoradiated (dark controls), and preparations not exposed to Photofrin II that were photoradiated (light controls). Enzyme activities in these controls did not vary $\pm 10\%$ from initial values.

*Enzyme activity analysis*

The activities of cytochrome c oxidase and succinate dehydrogenase (SDH) were analysed as previously (Gibson & Hilf, 1983; Hilf *et al.,* 1984). Control activities (before photoradiation) ranged from 0.4 to 0.6 $\mu$mol cytochrome c oxidised per min per mg protein for cytochrome c oxidase and from 4.6 to $8.3\times10^{-2}\,\mu$mol INT oxidised per min per mg protein for SDH.

NADH dehydrogenase activity was measured by the method of King & Howard (1962): 30 $\mu l$ of mitochondrial suspension were used and enzyme activity was assayed by monitoring, in a spectrophotometer at 420 nm, the reduction of ferricyanide. Enzyme activity was converted to equivalents of NADH oxidised per min; control values (before irradiation) ranged from 1.75 to 2.62 $\mu$mol NADH oxidised per min per mg protein.

The catalytic activity of $F_0F_1$ ATP synthase was analysed in sonicated mitochondrial suspensions (five 30 s periods of sonication on ice using a Biosonic III probe sonicator, adjusted to a setting of 35; Bronwill Scientific, Rochester, NY). Briefly, 30 $\mu l$ of the sonicated mitochondrial suspension (approximately 200 $\mu g$ protein per ml) was added to 1 ml of reaction mixture containing 50 mM Tris, pH 8.5, 10 mM ATP and 4 mM $MgCl_2$ to obtain the total ATPase activity; concurrently, a separate 30 $\mu l$ of sonicated mitochondrial suspension was added to the above reaction mixture containing, in addition, $25\,\mu g\,ml^{-1}$ of oligomycin to determine the oligomycin-sensitive ATPase activity. The difference between total and oligomycin-sensitive activity provides a measure of the catalytic activity of the $F_0F_1$ ATP synthase (in intact, non-sonicated, mitochondria the enzyme would utilise ADP and Pi to form ATP whereas, in the assay used here, it catalyses the reverse reaction, ATP → ADP + Pi). The reactions were incubated for 45 min at 37°C in a shaking water bath (New Brunswick Scientific, New Brunswick, NJ), tubes containing the samples were removed and 1 ml of 10% sodium dodecyl sulphate (SDS) was added to terminate the reactions. The amount of Pi released was analysed (Taussky & Shorr, 1953). The activity of the $F_0F_1$ ATPase ranged from $2.3\times10^{-2}$ to $4.3\times10^{-2}\,\mu$mol Pi released per min per mg protein (mean $3.0\times10^{-2}$).

Pyruvate kinase, an enzyme located in the cytosol, was assayed using 20 $\mu l$ aliquots of cytosols prepared from 10% tumour homogenates, according to methods described earlier (Hilf *et al.,* 1965). Control activities (analysis before photoradiation) were $0.742\pm0.096\,\mu$mol NADH oxidised per min per mg protein. All incubations required to determine enzyme activity were performed in the dark.

*Data analysis*

Data obtained for the effects of porphyrin photosensitisation are expressed as percentage of initial activity (zero time), the activity determined on samples before exposure to photoradiation. Rates of inhibition of enzyme activity were calculated from regression analysis of the linear portion of the inhibition curves. Results are presented as the mean ± s.e.m.

### Results

*Photosensitisation of mitochondrial NADH dehydrogenase by Photofrin II* in vitro

Before undertaking the *in vivo–in vitro* protocol study, we investigated whether Photofrin II could photosensitise mitochondrial NADH dehydrogenase *in vitro*. This enzyme is located in the inner membrane of mitochondria and functions to catalyse the reduction of ferricyanide, menadione, cytochrome c and coenzyme Q, constituents of Complex I of the respiratory chain. The data obtained in these experiments are illustrated in Figure 1. Photofrin II-induced photosensitisation of mitochondrial suspensions was manifested as a dose- and fluence-dependent inhibition of NADH dehydrogenase activity *in vitro*. The inset in Figure 1 depicts the rate of inhibition of NADH dehydrogenase at each concentration of Photofrin II used (0.7, 3.5, 7.0 and 35.0 $\mu$g/ml). The data depict a reasonably linear relationship between inhibition of NADH dehydrogenase activity and the Photofrin II concentration used up to $7.0\,\mu g\,ml^{-1}$; above this dose, however, linearity was lost. Thus, the activity of NADH dehydrogenase in isolated mitochondria could be inhibited by 50–60% by the higher concentrations of Photofrin II plus light *in vitro*.

*Effects of Photofrin II administered* in vivo *on photosensitisation of enzyme activity* in vitro

After administration of $25\,mg\,kg^{-1}$ Photofrin II to tumour-bearing rats, tumours were obtained at selected times for study of the effects of photoradiation *in vitro* on enzymes in mitochondria and cytosols. Results of the time-course of these responses in samples obtained from 30 min to 168 h after drug administration are illustrated in Figure 2. The pattern of responses appears to fall into three categories, based on the extent of inhibition observed. Pyruvate kinase, which is localised in the cytosol, displayed little or no inhibition of activity over the entire time course. NADH-

**Figure 1** Photofrin II concentration and fluence relationships for the inhibition of the activity of mitochondrial NADH dehydrogenase *in vitro*. Mitochondria, prepared from tumours of untreated rats as described in **Materials and methods**, were exposed to various concentrations of Photofrin II; $0.7\,\mu g\,ml^{-1}$ (○), $3.5\,\mu g\,ml^{-1}$ (●), $7.0\,\mu g\,ml^{-1}$ (□) and $35\,\mu g\,ml^{-1}$ (■). NADH dehydrogenase activity was analysed at various times during photoradiation (conditions detailed in **Materials and methods**). Data are presented as percentage of initial enzyme activity (zero time before light exposure). Each data point represents the mean of at least six separate experiments performed in duplicate. Error bars are the s.e.m. The inset represents the calculated rates of enzyme inhibition (% inhibition per $J\,cm^{-2}$) in relation to the Photofrin II concentration.

dehydrogenase activity, which was inhibited maximally to 35% at 24 h after Photofrin II administration, displayed inhibition of activity approximating 20% of control activity up to the 96 h time point. The other inner membrane mitochondrial enzymes, cytochrome c oxidase, succinate dehydrogenase and $F_0F_1$ ATPase, displayed the greatest inhibition of activity and all showed a similar pattern over the time course studied. Maximum inhibition of activity was observed when mitochondria were irradiated at 24–72 h after injection of Photofrin II. However, even at the latest time point examined, 168 h, photo-induced inhibition of enzyme activity (40–50%) was still demonstrable. These results demonstrate that certain mitochondrial enzymes located in the inner mitochondrial membrane are highly susceptible to photosensitisation by Photofrin II in this *in vivo–in vitro* protocol, whereas pyruvate kinase, an enzyme located in the cytosol, was virtually unaffected throughout the time course studied.

*Relationship of administered dose of Photofrin II on photo-induced inhibition of mitochondrial enzymes*

We next investigated whether the observed photosensitisation of mitochondrial enzymes was directly related to the administered dose of Photofrin II. The 24 h time point subsequent to administration of Photofrin II was selected, since this appeared to be the earliest time when maximal enzyme inhibition was obtained. The Photofrin II doses employed were 2.5, 5.0, 10.0 and $25\,mg\,kg^{-1}$; the results are depicted in Figure 3. The data demonstrate that each of the four mitochondrial enzymes studied displayed a dose- and fluence-dependent inhibition of activity. From these data, a rate of enzyme inhibition was calculated (% inhibition per $J\,cm^{-2}$) for each dose of Photofrin II administered *in vivo*. The rates were obtained by regression analysis of the initial region of the inhibition curve up to $270\,J\,cm^{-2}$ total fluence. When % inhibition per $J\,cm^{-2}$ was plotted against drug dose ($mg\,kg^{-1}$), a linear relationship was generally obtained, with deviation from linearity occurring only at the highest drug

**Figure 2** Time course of photosensitisation of enzymes in preparations of R3230AC mammary tumours following *in vivo* administration of $25\,mg\,kg^{-1}$ Photofrin II. Preparation of mitochondria and cytosols are detailed in **Materials and methods**. Enzyme activities analysed were pyruvate kinase (▲), NADH dehydrogenase (■), succinate dehydrogenase (◇), cytochrome c oxidase (△) and $F_0F_1$ ATPase (□). Each data point represents the mean of at least four separate experiments (one tumour-bearing animal per experiment), each assay performed in duplicate. Data are presented as percentage of initial (zero time) activity. Error bars are the s.e.m.

**Figure 3** Relationship of Photofrin II dose *in vivo* and fluence on the inhibition of selected mitochondrial enzymes. Mitochondria were prepared from tumours 24 h after injection with various doses of Photofrin II; 2.5 mg kg$^{-1}$ (△), 5.0 mg kg$^{-1}$ (▲), 10.0 mg kg$^{-1}$ (□) and 25 mg kg$^{-1}$ (■). Each panel depicts the results obtained for assay of each enzyme at each dose of Photofrin II used; (**a**) cytochrome c oxidase; (**b**) $F_0F_1$ ATPase; (**c**) succinate dehydrogenase; (**d**) NADH dehydrogenase. Data are expressed as percentage of initial enzyme activity (zero time before photoradiation of mitochondria). Each data point represents the mean of at least three separate experiments (one tumour-bearing animal per experiment). Error bars are the s.e.m. The inset in each panel represents the calculated rate of enzyme inhibition in relation to the Photofrin II dose administered *in vivo*.

dose for SDH and NADH dehydrogenase (see insets in Figure 3). These relationships between rate of inhibition and drug dose exhibited correlation coefficients of $r = 0.97$ or greater. The slopes of these lines can provide an estimate of relative sensitivity to photosensitisation for each enzyme. The values, % inhibition per J cm$^{-2}$ × 10$^{-2}$ per mg administered dose of Photofrin II, are: cytochrome c oxidase, 0.98; $F_0F_1$ ATPase, 0.77; succinate dehydrogenase, 0.49; and NADH dehydrogenase, 0.26. These data indicate that there are differences in the susceptibility of inner mitochondrial enzymes to photosensitisation by Photofrin II. It is interesting that over the time course studied, NADH dehydrogenase was the least affected under the conditions used in this *in vivo–in vitro* protocol. The data also suggests that photoradiation would be most effective in causing inhibition of mitochondrial enzymes when employed 24–72 h after administration of the photosensitiser.

### Discussion

Clinical treatment of malignancy by photodynamic therapy (PDT) holds considerable promise but optimisation of treatment, e.g. selection of sensitiser dose, total light fluence and timing, and elucidation of the mechanism(s) that result in retention of these porphyrin species for longer periods in tumour tissue than most normal tissues, are unresolved. Although studies of photosensitisation *in vitro* with any sensitiser, including metallophthalocyanines, kryptocyanines, rhodamines, etc., may provide useful data on selected parameters, the results may not be readily translatable to therapy *in vivo*. Therefore, we examined Photofrin II as we did previously for HpD (Hilf *et al.*, 1984), utilising an *in vivo–in vitro* protocol to investigate the effects of photosensitisation on discrete biochemical parameters. This protocol allows for metabolism of the sensitiser by the host, events that may influence equilibration of the sensitiser in neoplastic cells *in vivo*. After accounting for appropriate controls, a demonstration of photosensitisation by *in vitro* photoradiation of tumour preparations leads to the conclusion that a photosensitiser must have been present in the preparation under study.

The data presented provide the temporal pattern of Photofrin II-induced photosensitisation based on its capability to inhibit selected mitochondrial and cytosolic enzymes. These results clearly indicate that the greatest extent of light-induced inhibition of mitochondrial enzyme activity occurred between 24 and 72 h after administration of Photofrin II *in vivo*, a result quite comparable to that observed earlier when HpD was used as the photosensitiser (Hilf *et al.*, 1984). The results obtained for the cytosolic enzyme pyruvate kinase, however, were different when comparing HpD and Photofrin II in this *in vivo–in vitro* protocol. Administration of HpD resulted in an early and dramatic inhibition of pyruvate kinase activity (30 min to 24 h post-injection) when those tumour cytosols were exposed to visible light, but after 24 h pyruvate kinase activity was no longer sensitised to light by HpD. However, the results presented here using Photofrin II show that pyruvate kinase activity was unaffected throughout the time course studied. The apparent disparity is probably attributable to the different compositions of the two porphyrin preparations. HpD, prepared by the method of Lipson *et al.* (1960), is a complex mixture consisting of numerous hydrophilic and hydrophobic species, estimated previously to be 75% and 25% of the total porphyrins, respectively (Hilf *et al.*, 1983). The more hydrophilic porphyrin species of HpD, such as the isomers of haematoporphyrin and hydroxyethylvinyldeuteroporphyrin, were probably present in sufficient levels in the cytosol soon after injection such that exposure to light caused oxidative damage to cytosolic proteins. On the other hand, Photofrin II, a mixture of porphyrins enriched in the hydrophobic species (80–90% as reported by Dougherty (1987)), presumably dihaematoporphyrin ethers and/or esters (Berenbaum *et al.*, 1982; Byrne *et al.*, 1987; Dougherty, 1987; Kessel *et al.*, 1987), would be expected to accumulate primarily in the more hydrophobic regions, such as cell membranes. If this were the case, the lower concentration of hydrophilic components (20%) in Photofrin II would be less able to produce sufficient $^1O_2$ in the cytosol to cause inhibition of pyruvate kinase, the result we observed. Alternatively, the binding of those hydrophilic components in Photofrin II may not have been sufficient to maintain a porphyrin–pyruvate kinase complex for longer periods of time *in vivo*, as was suggested by Freitas & Novarina (1987) for lactate dehydrogenase in HeLa cells. It should be noted that we administered 80 mg kg$^{-1}$ HpD previously (Hilf *et al.*, 1984) against 25 mg kg$^{-1}$ Photofrin II in the present study and obtained similar patterns of inhibition of mitochondrial inner membrane enzymes. A simple calculation shows that similar amounts of hydrophobic porphyrin components were administered in both studies, approximately 20 mg kg$^{-1}$ from each preparation.

It is of continued interest that apparent differences exist in the photosensitivities of mitochondrial enzymes subsequent to administration of Photofrin II. From studies of enzymes *in vitro*, such differences did not appear to be attributable to intrinsic properties, since inhibitions of semi-purified enzymes by $^1O_2$ generation were comparable (Gibson *et al.*, 1987). Rather, we suggest that such differential sensitivity probably reflects differences in the immediate environment, their three-dimensional structure *in situ* and/or partitioning of hydrophobic porphyrins. Under the conditions studied here, the order of photosensitivity was cytochrome c oxidase $>F_0F_1$ ATPase >succinate dehydrogenase >NADH dehydrogenase. Cytochrome c oxidase, however, may possess some intrinsic properties that render it more sensitive to damage induced by porphyrin photosensitisation, such as the presence of hydrophobic regions where porphyrins may accumulate, since subunits I, II, III and VII display binding of hydrophobic reagent probes (DeMeis *et al.*, 1988). Also, it has been proposed that the haems and coppers may be bound to subunits I and II, although subunits V and VII have also been implicated as haem binding sites (Azzi, 1980). Likewise it appears that the active site of mitochondrial $F_1$ ATPase, and of the $Ca^{2+}$-ATPase of the sarcoplasmic reticulum, is hydrophobic in nature, since both enzymes were inhibited by hydrophobic drugs, an effect reversed by the presence of organic solvents (DeMeis *et al.*, 1988). Taken together, the greater sensitivities of cytochrome c oxidase and $F_0F_1$ ATPase to photosensitisation by Photofrin II may be attributed to partitioning of the hydrophobic porphyrin species in or near the active sites of these enzymes. If this were the case, generation of $^1O_2$ from illumination could have significant consequences.

Surprisingly little has been reported regarding the subcellular distribution of Photofrin II. A number of recent reports indicate that mitochondrial damage ensues photosensitisation by Photofrin II (Singh *et al.*, 1987; Moreno *et al.*, 1987), HpD (Berns *et al.*, 1982) and haematoporphyrin (Salet *et al.*, 1983). The data obtained here demonstrate the existence of a drug-dose related response of mitochondrial photosensitisation to Photofrin II. From these data, an optimum response to illumination should occur at 24–48 h after drug administration, assuming that the biochemical effects on mitochondria are important for subsequent cytotoxicity. This does not imply that other events affected by PDT, such as effects on vascularity (Fingar & Henderson, 1987; Selman *et al.*, 1985; Star *et al.*, 1986), are less important in producing tumour cell necrosis in the treated lesions. However the mechanisms for vascular cell damage might also involve similar cellular sites of action. An additional consideration is the expected hydrophobic nature of the inner mitochondrial membrane, which not only should favour accumulation of hydrophobic components of Photofrin II, but could also enhance the lifetime of $^1O_2$ (Parker & Stanbro, 1981), thus increasing the potential damage.

The data presented here, along with our earlier observations (Hilf *et al.*, 1984; Murant *et al.*, 1987; Gibson *et al.*, 1988), allow us to propose a chronology of intracellular distribution of photosensitising components of Photofrin II in a neoplasm *in vivo* (Table I). At 2 h after administration of Photofrin II, porphyrins have accumulated in the plasma membrane and have entered the outer membrane of mitochondria. This is deduced from the photoradiation-induced inhibition of $Na^+K^+$ATPase (plasma membrane) and monoamine oxidase (outer mitochondrial membrane) at 2 h post-treatment. At this early time after Photofrin II administration, as well as throughout the time-course studied, neither the cytosolic enzyme pyruvate kinase nor adenylate kinase, located in the intramembrane space of mitochondria, were inhibitable by photoradiation *in vitro*. Although photoradiation-induced inhibition of the inner mitochondrial membrane enzymes, succinate dehydrogenase, cytochrome c oxidase and $F_0F_1$ ATPase, was seen by 2 h post-treatment, the degree of inhibition of these enzymes progressively increased to reach a maximum by 24 h (Figure 2 and Table I). At the 24 h time point, only the inner mitochondrial membrane enzymes and the plasma membrane enzyme $Na^+K^+$ATPase demonstrated a significant inhibition. This photosensitisation persisted for 72 h, after which less inhibition resulted from illumination. Thus, if the effects observed on enzyme activity accurately reflect the location of photosensitisers, the active components in

**Table I** Photosensitisation of site selected enzymes by Photofrin II in R3230AC mammary tumours

| Enzyme | Time after Photofrin II administration 2 h | 24 h |
|---|---|---|
| $Na^+K^+$ ATPase | 43.8±4.7 | 55.6±7.0 |
| $Mg^{2+}$ ATPase | 92.2±1.8 | 86.4±2.5 |
| 5′-nucleotidase | 100±3.4 | 99.3±3.4 |
| Pyruvate kinase | 94.5±3.1 | 93.8±2.1 |
| Monoamine oxidase | 71.8±6.4 | 86.4±5.5 |
| Adenylate kinase | 95.8±1.5 | 93.7±1.0 |
| Cytochrome c oxidase | 67.0±4.0 | 26.8±1.7 |
| $F_0F_1$ ATPase | 62.6±4.5 | 34.4±4.2 |
| Succinate dehydrogenase | 79.2±2.0 | 39.7±2.7 |
| NADH dehydrogenase | 99.5±2.2 | 72.6±1.7 |

Tumour-bearing rats were injected i.p. with 25 mg kg$^{-1}$ Photofrin II, mitochondria prepared from tumours at selected times and exposed to 300–400 J cm$^{-2}$ broad band irradiation (see **Materials and methods**). Data are presented as percentage of initial enzyme activity (zero time before photoradiation) ± s.e.m. Each number represents the mean of at least four separate experiments performed in duplicate. Data for $Na^+K^+$ ATPase, $Mg^{2+}$ ATPase and 5′-nucleotidase are from Gibson *et al.* (1988) and for monoamine oxidase and adenylate kinase are from Murant *et al.* (1987).

Photofrin II demonstrate a time-dependent intracellular distribution that results in their retention in the plasma membrane and the inner mitochondrial membrane following *in vivo* administration. Bohmer & Morstyn (1985) reported that cellular uptake of HpD *in vitro* occurred in two phases; the first was rapid (seconds) and the porphyrin components were readily removed by washing with serum-containing medium, whereas the second phase took hours and the porphyrins incorporated into the cytosol and intracellular organelles could not be removed by serum-containing medium. A somewhat similar pattern was observed by Kessel (1986) with L1210 cells *in vitro* by comparing events after 30 min, 4 and 18 h incubations with HpD. Based on membrane transport, thymidine incorporation and cellular ATP levels, the longer incubation times resulted in photosensitised damage at intracellular membrane sites compared to the plasma membrane damage observed at the shorter incubation times. Earlier, Moan *et al.* (1983) observed that, in NHIK 3025 cells in culture, incubation with HpD for 1 versus 18 h resulted in a change of photosensitisable damage from the plasma membrane to intracellular sites as incubation times increased. The differences in the time course seen in our present study and those of Moan *et al.* or Kessel are probably attributable to differences in the exposure of tumour cells *in vivo* to circulating levels of porphyrins, extending the period of time that these cells and their organelles remain in the presence of the components of either HpD or Photofrin II.

In conclusion, administration of Photofrin II to tumour-bearing rats and study of the subsequent photosensitisation of selected enzymes, i.e. the *in vivo–in vitro* protocol, results in mitochondrial enzyme inhibitions that are dependent on the time interval between administration of the drug and exposure of the mitochondria to visible light, on the dose of Photofrin II administered *in vivo* and on the total fluence used to photoradiate the samples. Although the data presented here are in general agreement with data we previously obtained using HpD as the photosensitiser, clinical use of Photofrin II will require establishing a protocol to achieve maximum efficacy.

This study was supported by USPHS Grant CA36856, National Institutes of Health. We acknowledge the continued assistance of Kathy Faro and Kim Gabriel of the Animal Tumor Research Facility, University of Rochester Cancer Center (CA11198), for transplantation and maintenance of R3230AC mammary carcinoma. We appreciate the gift of Photofrin II from Photomedica Inc., Raritan, NJ and Quadra Logic Technologies Inc., Vancouver, BC, Canada, for these studies.

### References

AZZI, A. (1980). Cytochrome c oxidase: Towards a clarification of its structure, interactions and mechanism. *Biochim. Biophys. Acta*, **594**, 231.

BERENBAUM, M.C., BONNETT, R. & SCOURIDES, P.A. (1982). *In vivo* biological activity of the components of hematoporphyrin derivative. *Br. J. Cancer*, **45**, 571.

BERNS, M.W., DAHLMAN, A. & JOHNSON, F.M. & 8 others (1982). *In vitro* cellular effects of hematoporphyrin derivative. *Cancer Res.*, **42**, 2325.

BOHMER, R.M. & MORSTYN, G. (1985). Uptake of hematoporphyrin derivative by normal and malignant cells: Effect of serum, pH, temperature, and cell size. *Cancer Res.*, **45**, 5328.

BYRNE, C.J., MARSHALLSAY, L.V. & WARD, A.D. (1987). The structure of the active material in hematoporphyrin derivative. *Photochem. Photobiol.*, **46**, 575.

DEMEIS, L., TUENA DE GOMEZ PUYOU, M. & GOMEZ PUYOU, A. (1988). Inhibition of mitochondrial $F_1$ ATPase and sarcoplasmic reticulum ATPase by hydrophobic molecules. *Eur. J. Biochem.*, **171**, 343.

DOUGHERTY, T.J. (1987). Studies of the structure of porphyrins contained in Photofrin II. *Photochem. Photobiol.*, **46**, 569.

DOUGHERTY, T.J., POTTER, W.R. & WEISHAUPT, K.R. (1984). The structure of the active component of hematoporphyrin derivative. In *Porphyrin Localization and Treatment of Tumors*, Dorion, D.R. & Gomer, C.J. (eds) p. 301. Alan R. Liss: New York.

FINGAR, V.H. & HENDERSON, B.W. (1987). Drug and light dose dependence of photodynamic therapy: A study of tumor and normal tissue response. *Photochem. Photobiol.*, **46**, 837.

FREITAS, I. & NOVARINA, A. (1987). Dark effects of hematoporphyrin derivative on lactate dehydrogenase activity and distribution in HeLa cells: Cytochemical evaluation. *Photochem. Photobiol.*, **46**, 699.

GIBSON, S.L. & HILF, R. (1983). Photosensitization of mitochondrial cytochrome c oxidase by hematoporphyrin derivative and related porphyrins *in vitro* and *in vivo*. *Cancer Res.*, **43**, 4191.

GIBSON, S.L., COHEN, H.J. & HILF, R. (1984*a*). Evidence against the production of superoxide by photoirradiation of hematoporphyrin derivative. *Photochem. Photobiol.*, **40**, 441.

GIBSON, S.L., LEAKEY, P.B., CRUTE, J.J. & HILF, R. (1984*b*). Photosensitization of mitochondrial cytochrome c oxidase by hematoporphyrin derivative (HpD) *in vitro* and *in vivo*. In *Porphyrin Localization and Treatment of Tumors*, Dorion, D.R. & Gomer, C.J. (eds) p. 323. Alan R. Liss: New York.

GIBSON, S.L., MURANT, R.S. & HILF, R. (1987). Photosensitizing effects of hematoporphyrin derivative immobilized on sepharose. *Photochem. Photobiol.*, **45**, 93.

GIBSON, S.L., MURANT, R.S. & HILF, R. (1988). Photosensitizing effects of hematoporphyrin derivative and Photofrin II on the plasma membrane enzymes 5′-nucleotidase, $Na^+K^+$-ATPase and $Mg^{2+}$-ATPase in R3230AC mammary adenocarcinomas. *Cancer Res.*, **48**, 3360.

GOMER, C.J. & DOUGHERTY, T.J. (1979). Determination of [$^3$H] and [$^{14}$C] hematoporphyrin derivative distribution in malignant and normal tissue. *Cancer Res.*, **39**, 146.

HILF, R., MICHEL, I., BELL, C., FREEMAN, J.J. & BORMAN, A. (1965). Biochemical and morphological properties of a new lactating tumor line in the rat. *Cancer Res.*, **25**, 286.

HILF, R., LEAKEY, P.B., SOLLOTT, S.J. & GIBSON, S.L. (1983). Photodynamic inactivation of R3230AC mammary carcinoma *in vitro* with hematoporphyrin derivative: Effects of dose, time, and serum on uptake and phototoxicity. *Photochem. Photobiol.*, **37**, 633.

HILF, R., SMAIL, D.B., MURANT, R.S., LEAKEY, P.B. & GIBSON, S.L. (1984). Hematoporphyrin derivative-induced photosensitivity of mitochondrial succinate dehydrogenase and selected cytosolic enzymes of R3230AC mammary adenocarcinoma of rats. *Cancer Rats.*, **44**, 1483.

KESSEL, D. & CHOU, T.H. (1983). Tumor-localizing components of the porphyrin preparation hematoporphyrin derivative. *Cancer Res.*, **43**, 1994.

KESSEL, D., THOMPSON, P., MUSSELMAN, B. & CHANG, C.K. (1987). Chemistry of hematoporphyrin-derived photosensitizers. *Photochem. Photobiol.*, **46**, 563.

KING, T.E. & HOWARD, R.L. (1962). The preparation and some properties of a reduced diphosphopyridine nucleotide dehydrogenase from the snake venom digest of a heart muscle preparation. *J. Biol. Chem.*, **237**, 1686.

LIPSON, R.L., BALDES, E.J. & OLSEN, A.M. (1960). The use of a derivative of hematoporphyrin in tumor detection. *J. Natl Cancer Inst.*, **26**, 1.

MOAN, J., CHRISTENSEN, T. & SOMMER, S. (1982). The main photosensitizing components of hematoporphyrin derivative. *Cancer Lett.*, **15**, 161.

MOAN, J., McGHIE, J. & JACOBSEN, P.B. (1983). Photodynamic effects on cells *in vitro* exposed to hematoporphyrin derivative and light. *Photochem. Photobiol.*, **37**, 599.

MOAN, J., RIMINGTON, C. & SOMMER, S. (1987). Cellular uptake and photosensitizing properties of hematoporphyrin di-ethers with similar chromatographic properties as the tumor localizing fraction of hematoporphyrin derivative. *Cancer Lett.*, **34**, 283.

MORENO, G., ATLANTE, A., SALET, C. & 2 others (1987). Photosensitivity of DNA replication and respiration to hematoporphyrin derivative (Photofrin II) in mammalian CV-1 cells. *Int. J. Radiat. Biol.*, **52**, 213.

MURANT, R.S., GIBSON, S.L. & HILF, R. (1987). Photosensitizing effects of Photofrin II on site-selected mitochondrial enzymes: Adenylate kinase and monoamine oxidase. *Cancer Res.*, **47**, 4323.

PARKER, J.G. & STANBRO, D. (1981). Energy transfer processes accompanying laser excitation of hematoporphyrin in various solvents. *Johns Hopkins APL Digest*, **2**, 196.

PARKER, J.G. (1987). Optical monitoring of singlet oxygen generated during photodynamic treatment of tumors. *IEEE Circuits and Devices Magazine*, Jan. 10.

SALET, C., MORENO, G. & VINZENS, F. (1983). Effects of photodynamic action on energy coupling of $Ca^{2+}$ uptake in liver mitochondria. *Biochem. Biophys. Res. Comm.*, **115**, 76.

SELMAN, S.H., MILLIGAN, A.J., GOLDBLATT, P.J. & 3 others (1985). Correlation of tumor blood flow to tumor regression after hematoporphyrin derivative (HpD) photodynamic therapy to transplantable bladder tumors. *Adv. Exp. Med. Biol.*, **193**, 97.

SINGH, G., JEEVES, W.P., WILSON, B.C. & JANG, D. (1987). Mitochondrial photosensitization by Photofrin II. *Photochem. Photobiol.*, **46**, 645.

STAR, W.M., MARIJNISSEN, H.P.A., VANDENBERG BLOK, A.E. & REINHOLD, H.S. (1986). Destruction of rat mammary tumor and normal tissue microcirculation by hematoporphyrin derivative photoradiation observed *in vivo* in sandwich observation chambers. *Cancer Res.*, **46**, 2532.

TAUSSKY, H.H. & SHORR, E. (1953). A microcolorimetric method for the determination of inorganic phosphate. *J. Biol. Chem.*, **202**, 675.

WEISHAUPT, K.R., GOMER, C.J. & DOUGHERTY, T.J. (1976). Identification of singlet oxygen as the cytotoxic agent in photoinactivation of a murine tumor. *Cancer Res.*, **36**, 2322.